# Moderating role of triglycerides in the relationship between amyloid-β, hippocampal atrophy, and cognitive decline in mild cognitive impairment and early Alzheimer’s disease

**DOI:** 10.3389/fnagi.2025.1592341

**Published:** 2025-08-22

**Authors:** Ji-Woo Seok, Kahye Kim, Soonjo Hwang, Kun Ho Lee, Jaeuk U. Kim

**Affiliations:** ^1^Digital Health Research Division, Korea Institute of Oriental Medicine, Daejeon, Republic of Korea; ^2^Department of Psychiatry, University of Nebraska Medical Center, Omaha, NE, United States; ^3^Gwangju Alzheimer’s Disease & Related Dementias Cohort Research Center, Chosun University, Gwangju, Republic of Korea; ^4^Department of Biomedical Science, Chosun University, Gwangju, Republic of Korea; ^5^Dementia Research Group, Korea Brain Research Institute, Daegu, Republic of Korea; ^6^KM Convergence Science, University of Science and Technology, Daejeon, Republic of Korea

**Keywords:** triglycerides, amyloid-beta deposition, hippocampal atrophy, cognitive decline, moderated mediation

## Abstract

**Introduction:**

The role of triglycerides in Alzheimer’s disease dementia (ADD) progression remains unclear. This study aimed to investigate how triglyceride levels influence the relationship between amyloid-beta (Aβ) deposition, hippocampal atrophy, and cognitive decline in individuals with mild cognitive impairment (MCI) and early-stage ADD.

**Methods:**

A total 188 older adults (170 with MCI, 18 with early ADD) from the Gwangju Alzheimer’s Disease and Related cohort underwent amyloid PET and structural magnetic resonance imaging. Cognitive decline was assessed using the Korean version of the Mini-Mental State Examination (K-MMSE). A moderated mediation model (PROCESS model 7 with 10,000 bootstrap samples) was applied to examine whether hippocampal atrophy mediated the effect of Aβ on cognitive decline and whether this effect varied by triglyceride levels.

**Results:**

The indirect effect of Aβ deposition on cognitive decline through hippocampal atrophy was significant (bootstrap 95% CI: −0.39 to −0.08), while the direct effect was not. This suggests that hippocampal atrophy plays a substantial mediating role in the pathway from Aβ burden to cognitive decline, although the indirect path accounted for approximately 49% of the total effect, indicating the possibility of other unexplored mechanisms. Furthermore, moderated mediation analysis revealed that triglyceride levels significantly moderated the effect of Aβ deposition on hippocampal volume (*p* < 0.05), with higher triglyceride levels amplifying the negative impact of Aβ deposition on hippocampal atrophy.

**Discussion:**

These findings highlight hippocampal atrophy as a key pathway linking Aβ burden to cognitive impairment. Moreover, higher triglyceride levels may exacerbate Aβ-related neurodegeneration in individuals with MCI and early-stage ADD. Metabolic risk factors, such as triglycerides, may play an important role in strategies to prevent or delay cognitive decline in older adults with MCI and early ADD.

## 1 Introduction

Alzheimer’s disease dementia (ADD) is a globally prevalent neurodegenerative disorder primarily affecting adults aged > 65 years. Currently, over 55 million people worldwide live with dementia, with this number being projected to increase to 132 million by 2050 as a result of the aging population (World Health Organization, 2017). ADD accounts for approximately 60–70% of all dementia cases, making it the most common form of the condition (World Health Organization, 2017).

Recent research on ADD shifts focus from alleviating symptoms to developing “disease-modifying therapies” that intervenes or delays the underlying pathological mechanisms of the disease at its early stages (Hoffmann et al., 2011; Raghavan et al., 2013). These treatment strategies particularly target early intervention during the mild cognitive impairment (MCI) or preclinical ADD stages (Assunção et al., 2022). Since cognitive decline in early ADD and MCI progresses subtly and often remains clinically undetected (Samtani et al., 2012), elucidating the underlying pathophysiological mechanisms is crucial for informing effective prevention and early intervention strategies (Stephan et al., 2012). In this context, the Mini-Mental State Examination (MMSE) is widely used as a standard tool for assessing cognitive decline, evaluating various cognitive domains, such as memory, attention, orientation, and language (Folstein et al., 1975). Global cognition scores derived from the MMSE serve as a core clinical indicators that comprehensively reflect an individual’s overall cognitive function across multiple domains (Arevalo-Rodriguez et al., 2015). Therefore, understanding how pathological markers, such as amyloid-beta (Aβ), affect global cognitive function is critical for elucidating the early pathophysiology of ADD and developing prevention and intervention strategies.

ADD pathogenesis involves the accumulation of Aβ plaques and formation of neurofibrillary tangles by hyperphosphorylated tau protein, leading to widespread neuronal damage and brain atrophy (Huang and Jiang, 2009; Kocahan and Doğan, 2017). Aβ, which is derived from the amyloid precursor protein, abnormally accumulates in the brains of patients with ADD, resulting in Aβ plaques that disrupt cell function and contribute to neurodegeneration (Insel et al., 2016). This accumulation particularly impacts regions crucially involved in memory, such as the hippocampus, leading to volume reduction over time (Jack et al., 2015). The hippocampus is essential for memory formation and spatial navigation (Ekstrom and Hill, 2023). In patients with ADD, progressive Aβ deposition in the hippocampus and related structures, such as the entorhinal cortex, is correlated with early cognitive deficits (Devanand et al., 2007). This triggers pathological events, including synaptic dysfunction, neuroinflammation, and oxidative stress, leading to the degeneration of hippocampal neurons and hippocampal atrophy (Rao et al., 2022). Aβ levels are positively correlated to hippocampal volume loss, which is a hallmark of ADD progression (Rao et al., 2022).

Aβ deposition is influenced by both non-modifiable genetic risk factors, such as Apolipoprotein E (APOE) ε4 gene, and modifiable factors, including plasma cholesterol levels, which are associated with the risk of cognitive decline and ADD (Sáiz-Vazquez et al., 2020). However, epidemiological studies have reported inconsistent findings regarding the association between cholesterol levels and ADD-related cognitive decline (Anstey et al., 2017; Gong et al., 2022; Zhou et al., 2023). A recent analysis of data from more than five hundred thousand UK Biobank participants between 40 and 69 (mean: 56.5) years identified a U-shaped relationship between low-density lipoprotein (LDL), high-density lipoprotein (HDL), and total cholesterol levels with the risk of dementia, including vascular dementia and ADD (Gong et al., 2022). Contrastingly, an inverse relationship exists between triglyceride levels and the risk of ADD over a 12-year follow-up period (Gong et al., 2022). Unlike cholesterol levels, triglyceride levels are less affected by wasting diseases, including sarcopenia, and age-related factors that reduce cholesterol levels in later life (Zhou et al., 2023). This relative stability may partly explain why cholesterol levels show a U-shaped association with ADD risk, making them harder to interpret in older populations (Li et al., 2024). Therefore, triglyceride levels could serve as a more reliable biomarker for ADD risk in older populations (Zhou et al., 2023).

Triglycerides in the brain are essential for the synthesis and maintenance of neuronal cells, and they play a significant role in brain health (Tracey et al., 2018; Roy and Tedeschi, 2021). Additionally, they are critically involved in Aβ synthesis, deposition, and clearance (Yin, 2023). Although triglyceride levels are recognized as key atherogenic and ADD risk factors, reports have been inconsistent regarding the relationship between triglyceride levels and ADD risk (Solomon et al., 2007; Sáiz-Vazquez et al., 2020; Hong et al., 2021; Sun et al., 2023; Tian et al., 2023; Zhou et al., 2023). These inconsistencies may be attributed to the limited sample sizes of earlier studies (Nägga et al., 2018; Bennett et al., 2020), and the absence of comprehensive investigations into the relationship between triglyceride levels and ADD biomarkers in older adults. Therefore, we applied a moderated mediation framework to examine how Aβ deposition contributes to cognitive decline in older adults, both directly and indirectly through hippocampal atrophy, and whether this pathway is moderated by triglyceride levels.

The study hypotheses were as follows: (1) higher Aβ deposition is associated with increased cognitive decline (direct mediation); (2) higher Aβ deposition is linked to increased hippocampal atrophy, which is associated with increased cognitive decline (indirect mediation); (3) triglyceride levels moderate the effects of Aβ deposition on hippocampal atrophy, with higher triglyceride levels exacerbating hippocampal atrophy in older adults (moderated mediation); and (4) the moderating effect of triglyceride levels vary depending on their magnitude.

## 2 Materials and methods

### 2.1 Participants

We enrolled 188 individuals with cognitive decline (18 with ADD and 170 with MCI) aged ≥ 60 years from the Gwangju Alzheimer’s Disease and Related Dementia (GARD) cohort. The exclusion criteria were as follows: (1) structural lesions detected on magnetic resonance imaging (MRI), such as cerebral infarction, intracranial hemorrhage, brain tumors, or hydrocephalus; (2) significant neurological or psychiatric disorders that could affect cognitive abilities; (3) abnormal laboratory findings on complete blood count, serum electrolytes, liver/kidney/thyroid function tests, vitamin B12, folate, or syphilis serology; (4) focal brain lesions detected on MRI, such as lacunar infarcts and white matter hyperintensity; (5) current use of psychoactive medications; (6) illiteracy, which limits the ability to administer standardized cognitive tests (e.g., MMSE); and (7) severe visual or hearing impairments.

All participants completed a comprehensive ADD evaluation, amyloid positron emission tomography (PET), and structural MRI. Extensive data were collected from the GARD cohort, including demographic characteristics, psychological assessments, cognitive performance, current medical status, medical history, and neuroimaging data from MRI and amyloid PET. Diagnoses were based on the Diagnostic and Statistical Manual of Mental Disorders, Fifth Edition (DSM-5) criteria, the presence of Aβ, and confirmed neurodegeneration based on PET and MRI data (Seo et al., 2021). The diagnostic criteria for MCI were a clinical dementia rating (CDR) score of ≥ 0.5 and adherence to previously established MCI criteria (Winblad et al., 2004). The diagnostic criteria for ADD dementia were a CDR score of ≥ 0.5, and meeting the DSM-5 criteria for dementia and the criteria for probable ADD proposed by the National Institute of Neurological and Communication Disorders and Stroke-Alzheimer’s Disease and Related Disorders Association (McKhann et al., 1984). In line with the Institutional Review Board of Chonnam University Hospital (CNUH-2019-279), participants provided written informed consent prior to study participation.

### 2.2 Measurements

Demographic and social characteristics, including age, sex, education level, marital status, and monthly income, as well as clinical information, such as current medications, medical history, and any neurological or psychiatric disorders, were obtained from participants and their caregivers.

Cognitive decline was assessed using the Korean version of the MMSE (K-MMSE) (Han et al., 2024). The MMSE is the most widely used screening tool due to its ease of administration and proven effectiveness in detecting ADD (Boustani et al., 2003). A K-MMSE has been standardized, and normative data have been established based on tests conducted with Korean older adults (Lee et al., 2002; Han et al., 2008). The K-MMSE evaluates various cognitive domains, including temporal orientation (5 points), spatial orientation (5 points), immediate memory (3 points), attention and calculation (5 points), delayed memory recall (3 points), language (8 points), and visuospatial abilities (1 point). The MMSE has a maximum score of 30, where higher scores reflect better global cognitive functioning (Han et al., 2008). Although MMSE scores may vary depending on age, education, and sex (Piccinin et al., 2013), a total score of 23 is generally used as the standard cut-off point for identifying cognitive impairment. Based on commonly accepted criteria, MMSE scores of 24–30, 18–23, and 0–17 indicate absence, mild, and severe cognitive impairment, respectively (Yu et al., 2021).

### 2.3 Blood and CSF sample collection and biochemical analysis

Blood and cerebrospinal fluid (CSF) samples were collected from all participants, and were immediately purified and stored in a deep freezer and nitrogen tank. Cholesterol levels, including LDL, HDL, and triglycerides, were measured using an enzymatic colorimetric test with a Modular D2400 analyzer (Roche Diagnostics, Basel, Switzerland).

### 2.4 MRI acquisition and processing

We acquired T1-weighted anatomical images using a three-dimensional magnetization-prepared rapid gradient-echo sequence (Skyra, Siemens; 20-channel head coil; MRAGE sagittal view; repetition time = 2,300 ms; echo time = 2.143 ms; inversion time = 900 ms; flip angle = 9°; field of view = 256 mm × 256 mm; matrix = 320 × 320; slice thickness = 0.8 mm).

We employed voxel-based morphometry with the Computational Anatomy Toolbox 12 (CAT12) for SPM model generation (CAT12).^1^ The anatomical scans underwent the following preprocessing steps (Gupta et al., 2019): (1) spatial normalization and diffeomorphic anatomical registration via exponentiated lie algebra (Tustison et al., 2010); (2) modulation to create a template based on the Montreal Neurological Institute (MNI 152) space, followed by segmentation into gray matter (GM), white matter (WM), and CFS using a unified tissue segmentation technique (Ashburner and Friston, 2005); and (3) smoothing with an 8-mm full-width-at-half-maximum Gaussian filter. Preprocessing produced normalized, modulated, and segmented smooth images with a 1.5 mm voxel size and dimensions of 121 × 145 × 121 for each tissue type (GM, WM, CSF). Hippocampal volume was assessed using an automated segmentation technique incorporating atlas-based segmentation and morphological opening (Khagi et al., 2021).

### 2.5 PET data acquisition

All participants underwent PET scanning for 90–100 min. Aβ accumulation was assessed using the Discovery STE PET-CT scanner (General Electric Medical Systems, Milwaukee, WI, USA). Participants received an intravenous injection of 303 MBq ± 20% (18F) Florbetaben. The preprocessing of the PET images was performed as previously described (Choi et al., 2023). [18F]-FBB PET images were motion-corrected and co-registered with T1-weighted MRI images using the SPM12 toolbox^2^ in MATLAB (R2018a, MathWorks, Natick, MA, USA). The standard uptake value ratio (SUVR) was calculated to quantify cortical amyloid burden in six predefined regions, including the lateral temporal area, anterior and posterior cingulate, frontal area, and lateral parietal area, with the whole cerebellum served as the reference region.

### 2.6 Statistical analysis

All statistical analyses were performed using IBM SPSS Statistics for Windows, version 26.0 (IBM Corp., Armonk, NY, USA). Descriptive statistics were calculated for socio-demographic characteristics, cholesterol levels, gene type, hippocampal volume ratio, Aβ accumulation, and general cognition. Pearson’s correlations were used to assess relationships among triglyceride levels, hippocampal volume ratio, Aβ accumulation, and general cognition. To examine the association of triglyceride levels with Aβ accumulation and neurodegeneration, we conducted multivariate linear regression with Aβ accumulation and triglyceride levels as predictors and hippocampal volume as the outcome, with adjustment for socio-demographic variables, such as age, sex, and education. Additionally, multivariate linear regression was conducted with Aβ accumulation, hippocampal volume, and triglyceride levels as predictors and global cognition as the outcome, with adjustment for socio-demographic variables.

We conducted a moderated mediation analysis using the PROCESS macro for SPSS version 26, developed by Hayes (2012). First, we used PROCESS model 4, which estimates a simple mediation model, to test whether neurodegeneration (i.e., hippocampal volume ratio) mediates the relationship between Aβ deposition and global cognition (i.e., MMSE score). Next, we applied PROCESS model 7, which examines a moderated mediation model in which the effect of the independent variable on the mediator is moderated by a third variable. In our model, triglyceride levels were specified as the moderator of the relationship between Aβ accumulation and neurodegeneration (Hayes, 2012). Model 7 estimates conditional indirect effects by including a product term between the independent variable (i.e., Aβ deposition) and the moderator (i.e., triglyceride levels), thereby examining how the mediation process varies depending on triglyceride levels (Hayes, 2012). The model uses ordinary least squares to estimate mediation and moderated mediation effects, as well as bootstrapping to calculate 95% confidence intervals (CIs) for the indirect effects. Specifically, bias-corrected bootstrap CIs are derived from 10,000 samples at a 95% CIs. If the bootstrapped CIs do not include zero, the effect is considered significant (Hayes, 2012). All models were adjusted for sex, age, and education.

Additionally, to account for potential confounding effects of diagnosis group (ADD vs. MCI), we conducted a supplementary analysis with diagnosis as an additional covariate along with age, sex, and education.

## 3 Results

### 3.1 Participants characteristics

Table 1 presents the characteristics of the participants. A total of 188 older individuals with cognitive decline were included in the GARD cohort, comprising 170 individuals with MCI and 18 with early ADD. The average age of the participants was 73.95 years (standard deviation [SD] = 6.29), and 89 (47.30%) were female. The mean triglyceride level was 113.18 mg/dL (SD = 53.48), with a mean hippocampal volume of 7,345.56 mm^3^ (SD = 1,197.72), and mean amyloid SUVR of 1.14 (SD = 0.22). The average K-MMSE score was −21.51 (SD = 3.20), and 135 participants (71.81%) were APOE-ε4 carriers.

**TABLE 1:** Demographic and clinical characteristics.

Variable	Total (*N* = 188)	MCI (*n* = 170)	ADD (*n* = 18)
**Sex, *n* (%)**			
Male	89 (47.34)	84 (49.41)	5 (27.78)
Female	99 (52.66)	86 (50.59)	13 (72.22)
Age, years (mean ± SD)	73.95 ± 6.29	73.47 ± 6.15	78.50 ± 5.95
Education, years (mean ± SD)	14.12 ± 11.76	13.77 ± 10.41	17.44 ± 20.75
Height, cm (mean ± SD)	159.36 ± 10.23	159.21 ± 10.41	160.83 ± 8.47
Weight, kg (mean ± SD)	63.20 ± 13.51	63.08 ± 13.73	64.31 ± 11.55
BMI, kg/m^2^ (mean ± SD)	24.56 ± 3.24	24.54 ± 3.27	24.76 ± 2.99
Systolic BP, mmHg (mean ± SD)	124.21 ± 15.17	123.52 ± 15.16	130.72 ± 13.08
Diastolic BP, mmHg (mean ± SD)	70.89 ± 9.62	70.77 ± 9.67	72.06 ± 9.31
Glucose, mg/dL (mean ± SD)	80.67 ± 23.38	80.62 ± 23.74	81.17 ± 20.24
Total cholesterol, mg/dL (mean ± SD)	169.87 ± 38.13	170.42 ± 38.65	164.67 ± 33.24
HDL cholesterol, mg/dL (mean ± SD)	51.48 ± 14.20	51.63 ± 14.29	50.07 ± 13.54
LDL cholesterol, mg/dL (mean ± SD)	107.89 ± 34.24	108.26 ± 34.69	104.33 ± 30.40
Triglyceride, mg/dL (mean ± SD)	113.18 ± 53.48	113.6 ± 54.89	109.22 ± 38.54
*Gene type, *n* (%)			
E2/E2	0 (0)	0 (0)	0 (0)
E2/E3	4 (2.13)	4 (2.35)	0 (0)
E2/E4	4 (2.13)	3 (1.76)	1 (5.56)
E3/E3	49 (26.06)	44 (25.88)	5 (27.78)
E3/E4	117 (62.23)	106 (62.35)	11 (61.11)
E4/E4	14 (7.45)	13 (7.65)	1 (5.56)
GMV of hippocampus, (mean ± SD)	7,345.56 ± 1,197.72	7,499.92 ± 1,110.87	5,887.76 ± 1,011.82
Amyloid SUVR, (mean ± SD)	1.14 ± 0.22	1.13 ± 0.21	1.36 ± 0.19
K-MMSE scale, (mean ± SD)	21.51 ± 3.20	22.10 ± 2.27	15.97 ± 5.06

*Percentages may not total 100% due to rounding. ADD, Alzheimer’s disease; BMI, body mass index; BP, blood pressure; GDS, geriatric depression scale; GMV, gray matter volume; HDL, high-density lipoprotein; K-MMSE, Korean version of the Mini-Mental State Examination; NA, not applicable; LDL, low-density lipoprotein; MCI, mild cognitive impairment; SUVR, standardized uptake value ratio.

### 3.2 Correlation and multiple regression analyses

Aβ accumulation was negatively associated with triglyceride levels (*r* = −0.16, *p* < 0.05), global cognition (*r* = −0.25, *p* < 0.001), and hippocampal volume ratio (*r* = −0.39, *p* < 0.001). Conversely, the hippocampal volume ratio was positively correlated with global cognition (*r* = 0.36, *p* < 0.001).

The multiple regression model examining predictors of global cognition revealed that lower triglyceride levels (β = −0.17, *p* < 0.05) and greater hippocampal volume (β = 0.36, *p* < 0.001) were significantly associated with higher global cognition after adjusting for sex, age, and education. Additionally, hippocampal volume was significantly associated with Aβ accumulation (β = −0.33, *p* < 0.001), age (β = −0.46, *p* < 0.001), and sex (β = 0.26, *p* < 0.001) (Supplementary Tables 1, 2).

### 3.3 Mediation analysis

We initially investigated the potential mediating role of hippocampal volume by testing a regression model and calculating the indirect effect using PROCESS model 4, with sex, age, and education as covariates. As shown in Supplementary Table 3, Aβ accumulation was significantly negatively associated with hippocampal volume (β = −0.33, *p* < 0.001). Meanwhile, hippocampal volume significantly positively affected global cognition (β = 0.64, *p* < 0.001). However, Aβ accumulation had no direct effect on global cognition (β = −0.22, *p* = 0.11). The bootstrap analysis further confirmed a significant indirect effect of Aβ accumulation on global cognition via hippocampal volume (indirect effect = −0.21, 95% CIs: −0.38, −0.08) (Supplementary Table 4). The total effect of Aβ accumulation on global cognition was significant (β = −0.42, *p* < 0.01), and approximately 48.96% of this total effect was transmitted through the mediator (Proportion Mediated Effect, PME = 0.49). Collectively, these results suggest that hippocampal atrophy plays a substantial mediating role in the association between Aβ accumulation and global cognition, although the indirect pathway accounts for less than half of the total effect, indicating the potential involvement of additional mechanisms.

### 3.4 Moderated mediation analysis

**FIGURE 1:**
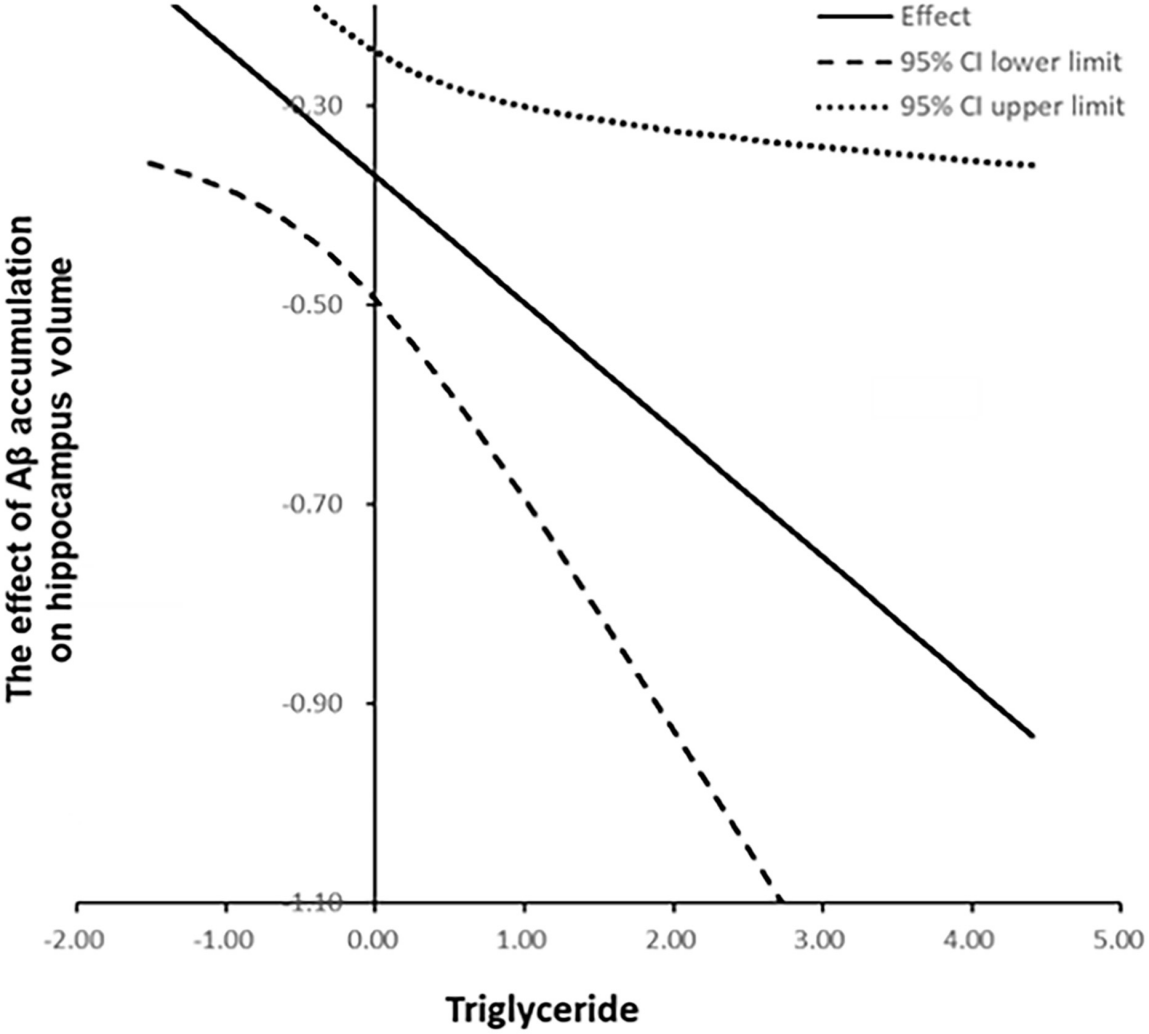
Moderated mediation model illustrating the indirect effect of amyloid-beta accumulation on global cognition, mediated by hippocampal gray matter volume and moderated by triglyceride levels.

We examined whether the observed mediated effects differed depending on triglyceride levels. As shown in Table 2 and Figure 1, triglyceride levels significantly moderated the relationship between Aβ accumulation and hippocampal volume (β = −0.13, *p* < 0.05), indicating that the strength of this association depends on triglyceride levels.

**TABLE 2:** Results of moderated mediation analysis including covariates.

IV	DV: GMV of hippocampal			DV: K-MMSE		
	β	SE	*t*	β	SE	*T*
Amyloid SUVR	−0.37	0.06	−5.89***	−0.22	0.13	−1.61
Sex	0.53	0.12	4.40***	−0.35	0.26	−1.34
Age	−0.07	0.01	−7.30***	0.02	0.02	1.03
Education year	−0.01	0.01	−1.80	−0.00	0.01	−0.44
Triglyceride	−0.03	0.06	−0.44	NA	NA	NA
Triglyceride * amyloid SUVR	−0.13	0.06	−2.14*	NA	NA	NA
GMV of the hippocampus	NA	NA	NA	0.64	0.16	4.11***
	R^2^ = 0.40, F = 19.87, *p* < 0.001			R^2^ = 0.15, F = 6.50, *p* < 0.001		

*p < 0.05,

***p < 0.001. IV, independent variable; DV, dependent variable; GMV, gray matter volume; K-MMSE, Korean version of the Mini-Mental State Examination; SUVR, standardized uptake value ratio; NA, not applicable; SE, standard error.

The impact of Aβ accumulation on hippocampal volume varied across different triglyceride levels (Supplementary Table 5). Notably, Aβ accumulation exerted a significant indirect effect on global cognition, mediated by hippocampal GMV reduction across all triglyceride levels, as the CIs did not include zero. At low triglyceride levels (59.97 mg/dL), Aβ accumulation reduced hippocampal GMV by −0.24 (95% CIs: −0.38, −0.10). At moderate triglyceride levels (113.45 mg/dL), this reduction was more pronounced at −0.37 (95% CIs: −0.50, −0.25). At a high triglyceride level (166.93 mg/dL), the reduction was most pronounced at −0.49 (95% CIs: −0.70, −0.30). Collectively, increased triglyceride levels exacerbate the negative impact of Aβ accumulation on hippocampal GMV.

**FIGURE 2:**
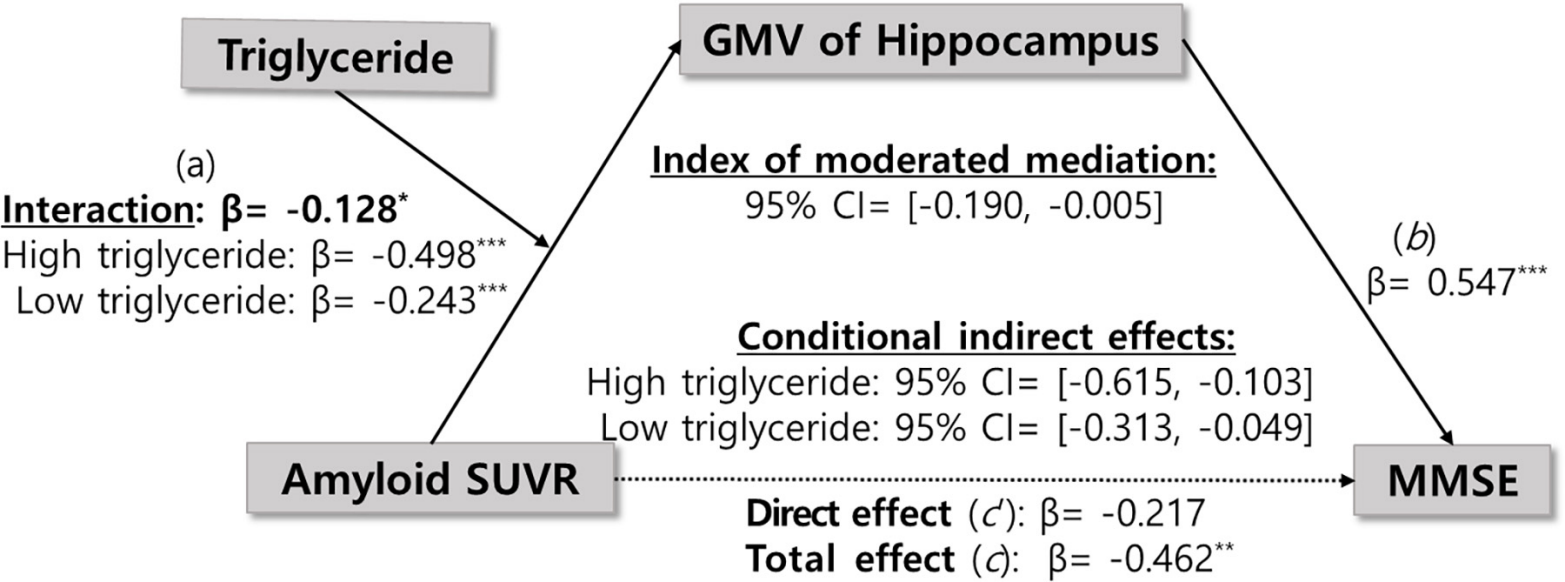
Distinct effects of triglyceride levels on the relationship between Aβ accumulation and hippocampal volume ratio. Aβ, amyloid-beta. Statistical significance is indicated by asterisks: *p < 0.05, **p < 0.01, ***p < 0.001.

**FIGURE 3:**
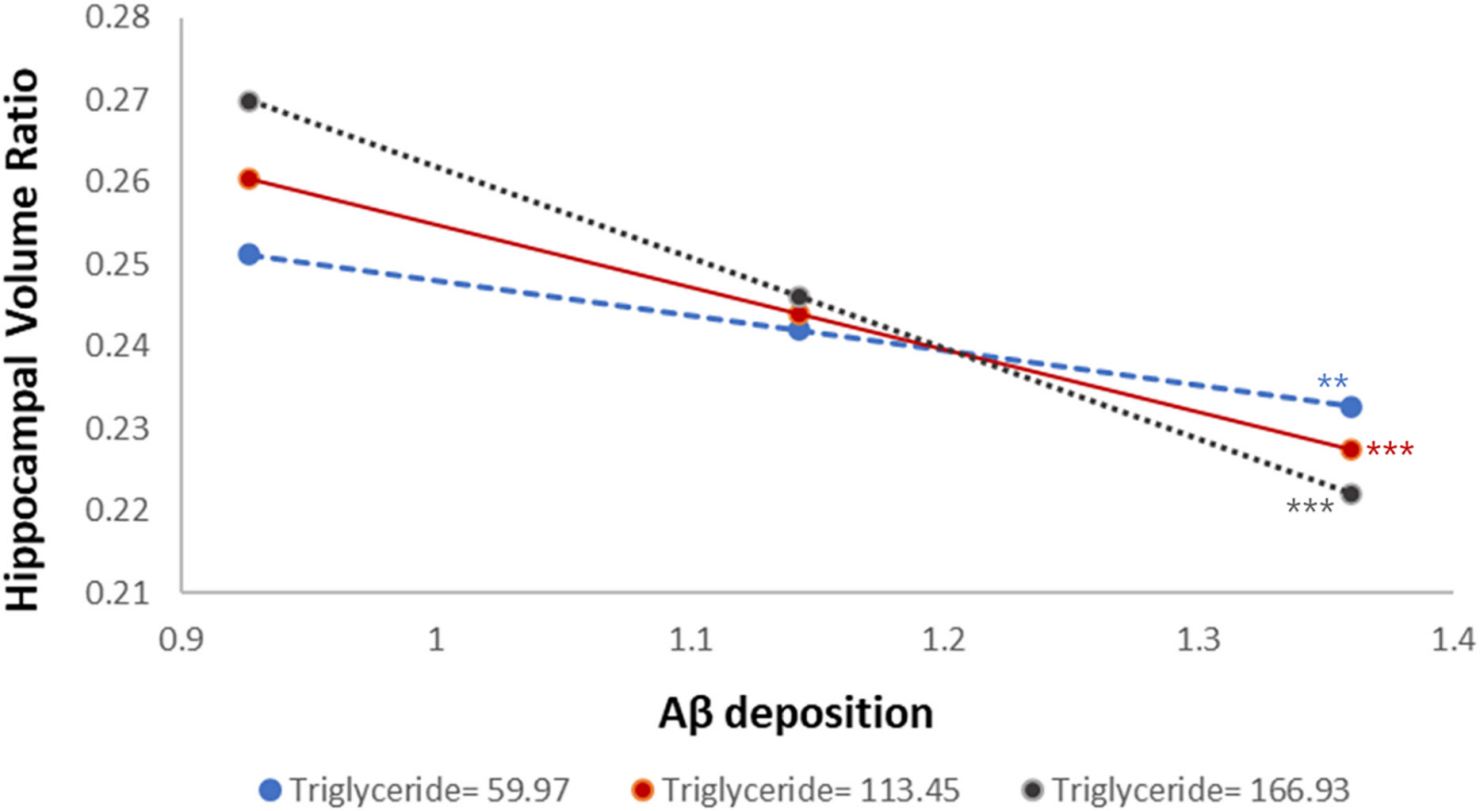
Johnson-Neyman plot illustrating the moderating effect of triglyceride levels on the association between amyloid-beta accumulation and hippocampal gray matter volume (dash-dot line with 95% confidence bands). The negative association remained significant across all triglyceride levels, as indicated by the solid vertical line. Statistical significance is indicated by asterisks: **p < 0.01, ***p < 0.001.

To better interpret the moderation effect, we illustrated the relationships by plotting values at one SD above and below the mean, representing high and low triglyceride levels, respectively (Figure 2). The Johnson–Neyman plot (Figure 3) illustrates the moderating effect of triglyceride levels on the relationship between Aβ accumulation and hippocampal volume. The *x*-axis represents triglyceride levels, while the *y*-axis shows the effect of Aβ accumulation on hippocampal volume. The solid line indicates the effect size, and the dashed lines represent the 95% CIs. The plot demonstrates that higher triglyceride levels exacerbate the negative impact of Aβ accumulation on hippocampal volume. The effect is significant across all triglyceride levels, as indicated by the solid vertical line.

In addition, the moderated mediation index was analyzed to evaluate whether the size of the indirect effect of Aβ accumulation on cognitive function varies significantly depending on triglyceride level. As shown in Table 3, the indirect effect was statistically significant at all three levels of triglycerides: low (59.97 mg/dL), mean (113.45 mg/dL), and high (166.93 mg/dL). The indirect effect was significant across all triglyceride levels, and the magnitude of the indirect effect gradually increased as the triglyceride level increased (low: β = −0.16, mean: β = −0.24, high: β = −0.32). This pattern indicates that higher triglyceride levels strengthened the indirect effect of Aβ accumulation on global cognition via hippocampal atrophy, suggesting that elevated triglyceride levels may exacerbate the cognitive consequences of Aβ-related neurodegeneration. However, the moderated mediation index, which assesses whether the strength of the indirect effect significantly differs across triglyceride levels, was not statistically significant (index = −0.08, 95% CIs: −0.20, 0.00). This indicates that although the indirect effect tended to increase with higher triglyceride levels, the variation was not statistically significant. In other words, although triglyceride levels moderated the pathway from Aβ accumulation to hippocampal atrophy, statistical evidence was insufficient to conclude that they significantly altered the overall indirect effect on global cognitive decline.

**TABLE 3:** Direct, conditional indirect, and moderated mediation effects in the model of Aβ accumulation, hippocampal volume, and global cognition (PROCESS Model 7).

Effect type	Estimate (β)	SE/Boot SE	*p*-value	95% CI	
				LLCI	ULCI
Direct effect	−0.22	0.13	0.11	−0.48	0.05
Indirect effect at low TG	−0.16	0.07	N.A.	−0.31	−0.05
Indirect effect at mean TG	−0.24	0.09	N.A.	−0.44	−0.09
Indirect effect at high TG	−0.32	0.13	N.A.	−0.62	−0.10
Index of moderated mediation	−0.08	0.05	N.A.	−0.20	0.00

Direct effect refers to the influence of Aβ accumulation on global cognition that is not transmitted through hippocampal volume. Indirect effects represent the pathway from Aβ accumulation to hippocampal volume to global cognition, calculated at three different levels of triglycerides: Low (−1 SD = 59.97 mg/dL), Mean (0 SD = 113.45 mg/dL), and High (+ 1 SD = 166.93 mg/dL). The index of moderated mediation tests whether the strength of the indirect effect significantly varies by triglyceride level.

In the adjusted analysis, which include the diagnosis group (ADD vs. MCI) as a covariate (Supplementary Table 6), Aβ accumulation had a significant negative effect on hippocampal volume (β = −0.29, *p* < 0.001), and hippocampal volume was significantly positively associated with cognitive function (β = 0.33, *p* < 0.05). However, triglyceride levels did not significantly moderate the association between Aβ accumulation and hippocampal volume (β = −0.10, *p* = 0.08), and the index of moderated mediation likewise failed to reach significance (95 CIs: −0.09, 0.01). Nonetheless, the indirect effects of Aβ on cognitive function through hippocampal volume were statistically significant at low (approximately 68 mg/dL), medium (approximately 98 mg/dL), and high (approximately 161 mg/dL) levels of triglycerides (Supplementary Table 7).

## 4 Discussion

This study analyzed the mediating effect of hippocampal atrophy on the relationship between Aβ accumulation and cognitive decline, as well as the moderating effect of triglyceride levels on this pathway. The results indicated that Aβ accumulation exerted an indirect effect on cognitive decline via hippocampal atrophy. This relationship was moderated by triglyceride levels, with hippocampal atrophy tending to worsen as triglyceride levels increased. However, the variation in the indirect effect by triglyceride levels was not statistically significant. These results suggest that triglycerides may contribute to hippocampal neurodegeneration associated with Aβ pathology.

The pathway by which Aβ deposition contributes to hippocampal atrophy and subsequent cognitive decline was proposed as a key process in the early stage of ADD pathology (Villeneuve et al., 2014; Mattsson et al., 2015). Previous studies have shown that Aβ deposition is associated with reduced cortical thickness and gray matter volume (GMV), which in turn mediate its effect on cognitive decline (Fletcher et al., 2018; Pelkmans et al., 2021). In line with these previous studies, the present study showed that hippocampal volume played a statistically significant mediating role in the relationship between Aβ deposition and global cognitive function. The direct effect of Aβ on cognition was not statistically significant, while the indirect effect via hippocampal atrophy was. Approximately 49% of the total effect was transmitted through the mediator (PME = 0.4896), indicating that although the mediation effect is meaningful, it accounts for less than half of the total association (Supplementary Table 4). This suggests that other potential mediating or moderating pathways may also contribute to the relationship between Aβ deposition and cognitive decline. A recent meta-analysis reported that hippocampal volumetry yielded sensitivity and specificity of 82% and 87% for ADD diagnosis, and 60% and 75% for MCI, respectively, supporting its value as a diagnostic biomarker (Park et al., 2022). These findings underscore the diagnostic and prognostic utility of hippocampal imaging as a neuroimaging marker for early ADD detection and evaluation of treatment effectiveness (Zhao et al., 2019).

Previous studies on the relationship between triglycerides and ADD have shown inconsistent results. A large-scale prospective cohort study that followed approximately 18,300 non-demented older adults aged ≥ 65 years found that individuals with higher triglyceride levels had a lower risk of ADD and experienced a significantly slower rate of cognitive decline over a median follow-up of 6.4 years (Zhou et al., 2023). In contrast, a longitudinal population-based study that followed 1,449 individuals aged ≥ 21 years found no significant association between late-life triglyceride levels and cognitive impairment or dementia risk (Solomon et al., 2007). Moreover, a meta-analysis reported that lipid components, such as LDL are more strongly related to ADD than triglycerides (Sáiz-Vazquez et al., 2020).

In this study, regression analysis showed no significant direct relationship between triglyceride levels and hippocampal gray matter volume (Supplementary Table 1). These discrepancies may be due to differences in the timing of lipid measurement or in the cognitive and pathological characteristics of the study populations. For instance, Zhou et al. (2023) assessed triglyceride levels in cognitively normal older adults and reported a protective effect, while Solomon et al. (2007) measured lipid levels in midlife and suggested that elevated triglycerides may serve as a long-term risk factor for dementia. Although both Zhou et al. (2023). and our study assessed triglycerides in late life, the pathological profiles of the participants differed substantially. Zhou et al. (2023) included healthy older adults, whereas our sample consisted of individuals already diagnosed with MCI or ADD, in whom substantial hippocampal atrophy and Aβ pathological accumulation are likely to be present. In this context, elevated triglyceride levels may no longer exert a protective effect, but rather contribute to disease progression.

Supporting this interpretation, our results show that the relationship between triglycerides and ADD is not uniform across all pathological states. In individuals with low Aβ accumulation (SUVR = 0.93), higher triglyceride levels were associated with greater hippocampal volume (Supplementary Figure 1), suggesting a potential protective role. In contrast, in those with high Aβ accumulation (SUVR = 1.36), higher triglyceride levels were linked to smaller hippocampal volume, implying a detrimental effect. These results indicate that triglycerides may act as a pathological context-dependent factor, exerting varying effects depending on the degree of Aβ burden. Our moderated mediation analysis further demonstrated that triglyceride levels significantly moderated the association between Aβ accumulation and hippocampal atrophy.

Notably, moderated mediation analysis revealed that triglyceride levels significantly moderated the relationship between Aβ accumulation and hippocampal volume (β = −0.13, *p* < 0.05), with the strength of the association varying by triglyceride levels. When triglyceride levels were low, the regression coefficient for the association between Aβ accumulation and hippocampal volume was −0.24, becoming more negative at −0.37 and −0.49 for intermediate and high triglyceride levels, respectively (Supplementary Table 5). This result shows that the negative association between Aβ accumulation and hippocampal volume strengthens with increasing triglyceride levels. The index of moderated mediation was also computed to evaluate whether this moderating effect extends to the entire indirect pathway from Aβ accumulation through hippocampal atrophy to cognitive decline. The indirect effect was significant at all triglyceride levels, increasing in magnitude from β = −0.16 at low levels, β = −0.24 at medium levels, and β = −0.32 at high levels. However, the index of moderated mediation was not significant (95% CI: −0.20, 0.00), indicating that the differences in the indirect effect across triglyceride levels were not statistically significant. These results suggest that triglycerides may moderate the relationship between Aβ accumulation and hippocampal atrophy. However, the evidence is insufficient to confirm that this moderation explains the full pathway to cognitive decline. Nevertheless, since the indirect effect remained significant across all triglyceride levels, their potential influence on Aβ-related neurodegeneration cannot be ruled out.

In the analysis adjusting for the diagnostic group (ADD vs. MCI), both the moderating effect of triglycerides (β = −0.10, *p* = 0.08), and the index of moderated mediation were not significant (Supplementary Table 6). However, even when the diagnostic group was controlled, the indirect effect of Aβ accumulation on cognitive function mediated by hippocampal volume was consistently significant at all triglyceride levels (Supplementary Table 7). These results suggest that triglyceride levels may exert a modulatory effect on the Aβ-related neurodegenerative pathway, warranting further investigation into the underlying biological mechanisms.

Growing evidence suggests that triglycerides may exert different effects in ADD pathology depending on Aβ accumulation and metabolic health, rather than functioning in a single direction (Reitz, 2013; Sun et al., 2023; Zhou et al., 2023). Recent studies indicate that triglycerides may influence hippocampal atrophy and cognitive decline via either neuroprotective or neurotoxic mechanisms (Bernath et al., 2020; Hong et al., 2021; Tian et al., 2023). When Aβ accumulation is low or the pathology is relatively mild, triglycerides may reflect better metabolic health or exert neuroprotective effects (Zhou et al., 2023). For example, individuals with lower levels of polyunsaturated fatty acid containing triglycerides (PUTG), a specific component of triglycerides, exhibited more severe atrophy in the hippocampus and medial temporal lobe, consistent with neuroimaging markers of early ADD (Bernath et al., 2020). These findings suggest that triglyceride components, such as PUTG may contribute to the preservation of hippocampal structures.

In contrast, when Aβ accumulation has already progressed to a significant level, triglycerides may exacerbate neurodegeneration. First, hypertriglyceridemia promotes the formation of atherosclerotic plaques in systemic and cerebral arteries, thereby reducing cerebral blood flow, which is a major risk factor for cognitive decline (Park et al., 2007; Peng et al., 2017; Dimache et al., 2021). Second, higher triglyceride levels may enhance inflammatory responses and oxidative stress, which can lead to neurodegenerative changes, such as mitochondrial dysfunction, synaptic loss, and hippocampal atrophy (Dimache et al., 2021; Yin, 2023). Third, circulating Aβ produced in intestinal epithelial cells can bind to triglyceride-rich lipoproteins, such as very low-density lipoproteins, increasing blood-brain barrier permeability and facilitating Aβ entry into the brain, thereby promoting its accumulation (Burgess et al., 2006; Takechi et al., 2008; Do et al., 2013; Banks et al., 2018). In addition, reduced activity of the peroxisome proliferator-activated receptor gamma receptor, a key factor regulating triglycerides metabolism, impairs lipolytic enzymes, and at weakens Aβ clearance, potentially accelerating Aβ accumulation in neurons (Kershaw et al., 2007; Dimache et al., 2021).

Recent studies demonstrated that astrocyte-mediated lipid metabolism and Aβ clearance are essential for maintaining hippocampal integrity (Cao et al., 2024). Astrocytes are a major glial cell population in the brain that synthesize and regulate cholesterol and fatty acids, and deliver them to neurons to support synaptic stability and neuronal survival (Heino et al., 2000; Cao et al., 2024). These functions are regulated by lipid metabolism-related genes, such as ATP-binding cassette transporter A1 (ABCA1), ATP-binding cassette transporter A7, and APOE, and dysfunction of these genes can lead to decreased ApoE lipidation efficiency and impaired Aβ clearance capacity (Moulton et al., 2021; von Maydell et al., 2024). Decreased ABCA1 function is linked to reduced circulating ApoE levels and increased ADD risk (Nordestgaard et al., 2015), while ABCA7 mutations are associated with disruptions in Aβ and lipid metabolism (Aikawa et al., 2018; De Roeck et al., 2019). Impaired lipid transport function can lead to Aβ accumulation, enhanced neuroinflammatory responses, synaptic degeneration, and other pathological changes that contribute to hippocampal dysfunction (Cao et al., 2024). Therefore, in this study, the tendency of elevated triglyceride levels to exacerbate Aβ-related hippocampal atrophy suggests a pathophysiological link between impaired lipid transport function in astrocytes and disturbances in Aβ metabolism, beyond a simple increase in peripheral lipid levels (Moulton et al., 2021; von Maydell et al., 2024; Aikawa et al., 2018; De Roeck et al., 2019; Nordestgaard et al., 2015).

This study has some limitations. First, the cross-sectional design precludes cause-and-effect relationships. Longitudinal studies are warranted to confirm these relationships. Second, although our sample is representative of older adults with cognitive decline, future studies should include more diverse populations, such as cognitively normal individuals and patients with severe ADD, to broaden generalizability. The limited number of ADD participants in our sample may have constrained the statistical power to identify group-specific effects, especially in moderation analyses related to diagnostic groups. Nevertheless, all indirect effects remained significant across varying triglyceride levels, indicating that triglycerides may influence the Aβ-related neurodegenerative pathway involving hippocampal atrophy and cognitive decline. Future research should employ larger and more balanced longitudinal cohorts to accurately investigate the influence of diagnostic group characteristics on this moderated mediation pathway. Third, participants with low literacy were excluded to ensure valid administration of standardized cognitive assessments, such as the MMSE, which may limit the generalizability of the findings to older adults with limited literacy. Future research should consider integrating literacy-independent tools, such as the Literacy Independent Cognitive Assessment (LICA) (Shim et al., 2015), which was specifically developed for use in this patient population. Fourth, future research should explore the underlying mechanisms linking genetic vulnerability, triglycerides, Aβ accumulation, and hippocampal atrophy. The correlation between triglycerides and ADD risk may vary among older adults based on their genetic susceptibility (e.g., the apolipoprotein A5-1131C allele variant), which has been linked to both elevated triglyceride levels and neurocognitive issues (Ancelin et al., 2013). However, evidence supporting this genetic interaction is limited. Additionally, investigating other lipid-related biomarkers may enhance our understanding of the metabolic contributions to ADD pathology. Fifth, moderated mediation analysis requires a sufficiently large sample size to reliably detect interaction effects. This study included a moderate-sized sample (*n* = 188), which may have limited the statistical power to detect interaction effects. Nevertheless, the indirect effect of Aβ accumulation on cognitive function via hippocampal atrophy was consistently significant across different levels of triglycerides, with moderation evident in specific pathways. These findings may support the overall reliability of the study’s results. Future research should aim to validate the stability and robustness of the moderated mediation effect using large-scale longitudinal datasets. Finally, sex-based moderated mediation model was not applied due to the small sample sizes of both male and female subgroups (< 100 participants each), which substantially reduced the statistical power to detect meaningful effects. Consequently, potential effects may gone undetected, limiting the generalizability of our findings regarding sex differences. Future studies should include large-scale cohorts to facilitate more robust analyses of sex-specific effects, and improve the generalizability of the results across different populations.

## 5 Conclusion

This study highlights a model of ADD pathology in which hippocampal atrophy serves as a substantial mediator linking Aβ accumulation to cognitive decline, with triglyceride levels potentially moderating this pathological pathway. Higher triglyceride levels were associated with a stronger impact of Aβ accumulation on hippocampal volume reduction, suggesting its contribution to the amplification of ADD pathology beyond their traditional role as a metabolic biomarker.

This study also suggests that inconsistencies in previous findings on the link between triglycerides and ADD may be attributed to differences in pathological context, such as the level of Aβ accumulation or diagnostic group (i.e., MCI vs. ADD). These findings provide valuable insights for guiding future research and designing clinical interventions targeting the role of triglycerides in ADD pathology. Importantly, these results underscore the need to account for individual pathological profiles when evaluating the impact of triglycerides on ADD progression, supporting more personalized approaches to prevention and intervention strategies.

## Data Availability

The raw data supporting the conclusions of this article will be made available by the authors, without undue reservation.

## Footnotes

1
http://dbm.neuro.uni-jena.de/cat/

2
https://www.fil.ion.ucl.ac.uk/spm/
