# Supplementary material for: Moderating role of triglycerides in the relationship between amyloid-β, hippocampal atrophy, and cognitive decline in mild cognitive impairment and early Alzheimer’s disease
Source: Front Aging Neurosci. 2025 Aug 22;17:1592341. doi: 10.3389/fnagi.2025.1592341 (PMC12411439; doi:10.3389/fnagi.2025.1592341)

Supplementary Material

1. **Supplementary Tables**

**Supplementary Table 1. Standardized coefficients from multiple regression predicting hippocampal gray matter volume**

|  | **B** | **SE** | β | **t** | **p value** |
| --- | --- | --- | --- | --- | --- |
| Triglyceride | -0.10 | 1.33 | -0.00 | -0.74 | 0.94 |
| Amyloid SUVR | -1804.25 | 331.93 | -0.33 | -5.44 | 0.00 |
| Sex | 613.44 | 144.47 | 0.26 | 4.25 | 0.00 |
| Age | -87.28 | 11.74 | -0.46 | -7.43 | 0.00 |
| Education | -10.03 | 5.95 | -0.10 | -1.69 | 0.09 |

Aβ, amyloid-beta; HVR, Hippocampus volume ratio; SE, Standard error; SUVR, Standardized uptake value ratio.

Unstandardized coefficients (B) represent the expected change in hippocampal gray matter volume for a one-unit change in each predictor. Standardized coefficients (β) indicate the change in hippocampal volume in standard deviation units, allowing comparison of the relative importance of predictors.

**Supplementary Table 2. Standardized coefficients from multiple regression predicting global cognition**

|  | **B** | **SE** | β | **t** | **p value** |
| --- | --- | --- | --- | --- | --- |
| Triglyceride | -0.01 | 0.00 | -0.17 | -2.51 | 0.01 |
| Amyloid SUVR | -1.20 | 0.62 | -0.15 | -1.94 | 0.05 |
| HVR | 0.00 | 0.00 | 0.36 | 4.15 | 0.00 |
| Sex | -0.39 | 0.26 | -0.11 | -1.48 | 0.14 |
| Age | 0.02 | 0.02 | 0.07 | 0.83 | 0.41 |
| Education | -0.01 | -0.01 | -0.03 | -0.50 | 0.62 |

Aβ, amyloid-beta; HVR, Hippocampus volume ratio; SE, Standard Error; SUVR, Standardized uptake value ratio.

Unstandardized coefficients (B) represent the expected change in the global cognition score (MMSE) for a one-unit increase in each predictor, expressed in original measurement units. Standardized coefficients (β) represent the change in the outcome variable in standard deviation units, enabling comparison of the relative importance of the predictors.

**Supplementary Table 3. Results of the mediation analysis, including covariates**

| **IV** | **DV: GMV of the hippocampus** | | | **DV: K-MMSE** | | |
| --- | --- | --- | --- | --- | --- | --- |
|  | **β** | **SE** | **t** | **β** | **SE** | **t** |
| Amyloid SUVR | -0.33 | 0.06 | -5.49^***^ | -0.22 | 0.13 | -1.61 |
| Sex | 0.51 | 0.12 | 4.27^***^ | -0.35 | 0.26 | -1.34 |
| Age | -0.07 | 0.01 | -7.48^***^ | 0.02 | 0.02 | 1.03 |
| Education year | -0.01 | 0.01 | -1.69 | -0.00 | 0.01 | -0.44 |
| GMV of the hippocampus | N.A. | N.A. | N.A. | 0.64 | 0.15 | 4.11^***^ |
|  | R^2^ = 0.38, F = 28.27, p < 0.001 | | | R^2^ = 0.15, F = 6.50, p < 0.001 | | |

*p<.05, **p<.01, ***p<.001

Aβ, amyloid-beta; IV, Independent variable; DV, Dependent variable; GMV, Gray Matter Volume; K-MMSE, Korean Version of the Mini-Mental State Examination; SUVR, Standardized uptake value ratio

**Supplementary Table 4. Mediation analysis results including total, direct, indirect effects and proportion mediated effect**

| **Effect Type** | **Estimate** | **SE/Boot SE** | **p-value** | **95% CI** | |
| --- | --- | --- | --- | --- | --- |
|  |  |  |  | **LLCI** | **ULCI** |
| Total effect (c) | -0.42 | 0.13 | 0.00 | -0.68 | -0.17 |
| Direct effect (c′) | -0.22 | 0.13 | 0.11 | -0.48 | 0.05 |
| Indirect effect (a × b) | -0.21 | 0.08 | N.A. | -0.38 | -0.08 |
| PME | 0.49 | N.A. | N.A. | N.A. | N.A. |

Values are based on a mediation analysis conducted using PROCESS macro Model 4 with 10,000 bootstrap samples and covariates (sex, age, and education) included.
The total effect (c) represents the overall effect of the independent variable (Aβ accumulation) on the dependent variable (global cognition).
The direct effect (c′) reflects the effect of Aβ accumulation on global cognition after accounting for the mediator (hippocampal volume).
The indirect effect represents the effect of Aβ accumulation on global cognition that operates through hippocampal volume.
The proportion mediated effect (PME) was calculated as the ratio of the indirect effect to the total effect.
95% CI, 95% confidence interval; SE, standard error; BootSE, bootstrap standard error; LLCI, The lower limit of the 95% confidence intervals; ULCI, The upper limit of the 95% confidence intervals.

**Supplementary Table 5. Moderator analysis: Conditional effect of Aβ accumulation on the hippocampal gray matter volume at values of the moderator (Triglyceride with Aβ accumulation).**

| **Triglyceride**  **Levels** | **Effect** | **Boot SE** | **95% CI** | |
| --- | --- | --- | --- | --- |
|  |  |  | **LLCI** | **ULCI** |
| 59.97 | -0.24 | .07 | -0.38 | -0.10 |
| 113.45 | -0.37 | .06 | -0.50 | -0.25 |
| 166.93 | -0.50 | .10 | -0.70 | -0.30 |

Boot LLCI: The lower limit of the 95% confidence intervals

Boot ULCI: The upper limit of the 95% confidence intervals

Aβ, amyloid-beta; CI, confidence interval.

**Supplementary Table 6. Results of moderated mediation analysis including diagnosis group (ADD vs. MCI), sex, and age as covariates.**

| **IV** | **DV: GMV of hippocampal** | | | **DV: K-MMSE** | | |
| --- | --- | --- | --- | --- | --- | --- |
|  | **β** | **SE** | **t** | **β** | **SE** | **t** |
| Amyloid SUVR | -.29 | .06 | -4.56^***^ | -.06 | .12 | -.49 |
| Sex | .56 | .12 | 4.86^***^ | -.05 | .24 | -.23 |
| Age | -.07 | .01 | -6.87^***^ | .02 | .02 | 1.20 |
| Diagnosis | -.80 | .21 | -3.86^***^ | -3.02 | .41 | -7.33^***^ |
| Education Year | -.01 | .00 | -1.38 | .00 | .01 | .11 |
| Triglyceride | -.01 | .06 | -.16 | NA | NA | NA |
| Triglyceride * Amyloid SUVR | -.10 | .06 | -1.73 | NA | NA | NA |
| GMV of the hippocampus | NA | NA | NA | .33 | .14 | 2.34^*^ |
|  | R^2^ = 0.44, F = 20.46,  p < 0.001 | | | R^2^ = 0.35, F = 15.94,  p < 0.001 | | |

*p < .05, **p < .01, ***p < .001

IV, Independent variable; DV, Dependent variable; GMV, Gray Matter Volume; K-MMSE, Korean Version of the Mini-Mental State Examination; SUVR, Standardized uptake value ratio; NA, Not Applicable; SE, standard error.

**Supplementary Table 7. Conditional effects of Aβ accumulation on hippocampal gray matter volume at representative triglyceride levels, adjusting for diagnosis group (ADD vs. MCI), sex, and age.**

| **Triglyceride**  **Levels** | **Effect** | **Boot SE** | **95% CI** | |
| --- | --- | --- | --- | --- |
|  |  |  | **LLCI** | **ULCI** |
| 65.75 | -0.21 | 0.07 | -0.34 | -0.08 |
| 97.74 | -0.26 | 0.06 | -0.38 | -0.14 |
| 161.24 | -0.38 | 0.10 | -0.57 | -0.19 |

Boot LLCI: The lower limit of the 95% confidence intervals

Boot ULCI: The upper limit of the 95% confidence intervals

Aβ, amyloid-beta; CI, confidence interval.

1. **Supplementary Figures**

**Supplementary Figure 1. Moderating effect of triglyceride levels on the relationship between Aβ deposition and hippocampal volume ratio**


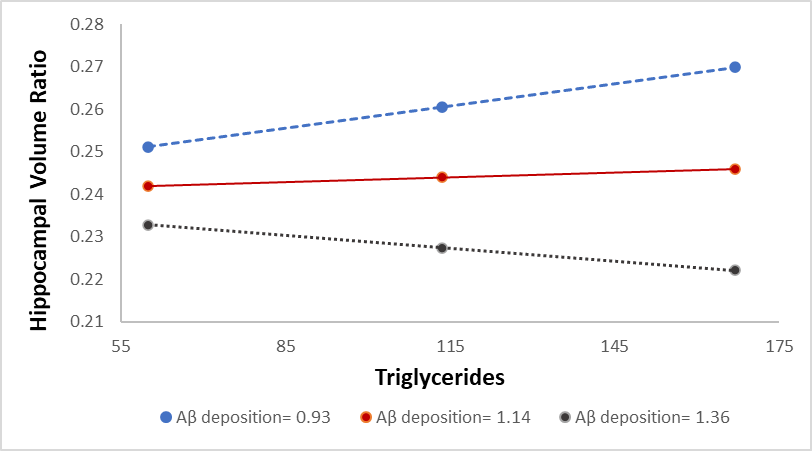

Supplement: Supplementary file 1 [file Data_Sheet_1.docx]
